# Developments in the Frequency of Ratings and Evaluation Tendencies: A Review of German Physician Rating Websites

**DOI:** 10.2196/jmir.6599

**Published:** 2017-08-25

**Authors:** Stuart McLennan, Daniel Strech, Swantje Reimann

**Affiliations:** ^1^ Institute for History, Ethics and Philosophy of Medicine Hannover Medical School Hannover Germany; ^2^ Institute for Biomedical Ethics Universität Basel Basel Switzerland

**Keywords:** physician rating websites, patient satisfaction

## Abstract

**Background:**

Physician rating websites (PRWs) have been developed to allow all patients to rate, comment, and discuss physicians’ quality online as a source of information for others searching for a physician. At the beginning of 2010, a sample of 298 randomly selected physicians from the physician associations in Hamburg and Thuringia were searched for on 6 German PRWs to examine the frequency of ratings and evaluation tendencies.

**Objective:**

The objective of this study was to examine (1) the number of identifiable physicians on German PRWs; (2) the number of rated physicians on German PRWs; (3) the average and maximum number of ratings per physician on German PRWs; (4) the average rating on German PRWs; (5) the website visitor ranking positions of German PRWs; and (6) how these data compare with 2010 results.

**Methods:**

A random stratified sample of 298 selected physicians from the physician associations in Hamburg and Thuringia was generated. Every selected physician was searched for on the 6 PRWs (Jameda, Imedo, Docinsider, Esando, Topmedic, and Medführer) used in the 2010 study and a PRW, Arztnavigator, launched by Allgemeine Ortskrankenkasse (AOK).

**Results:**

The results were as follows: (1) Between 65.1% (194/298) on Imedo to 94.6% (282/298) on AOK-Arztnavigator of the physicians were identified on the selected PRWs. (2) Between 16.4% (49/298) on Esando to 83.2% (248/298) on Jameda of the sample had been rated at least once. (3) The average number of ratings per physician ranged from 1.2 (Esando) to 7.5 (AOK-Arztnavigator). The maximum number of ratings per physician ranged from 3 (Esando) to 115 (Docinsider), indicating an increase compared with the ratings of 2 to 27 in the 2010 study sample. (4) The average converted standardized rating (1=positive, 2=neutral, and 3=negative) ranged from 1.0 (Medführer) to 1.2 (Jameda and Topmedic). (5) Only Jameda (position 317) and Medführer (position 9796) were placed among the top 10,000 visited websites in Germany.

**Conclusions:**

Whereas there has been an overall increase in the number of ratings when summing up ratings from all 7 analyzed German PRWs, this represents an average addition of only 4 new ratings per physician in a year. The increase has also not been even across the PRWs, and it would be advisable for the users of PRWs to utilize a number of PRWs to ascertain the rating of any given physician. Further research is needed to identify barriers for patients to rate their physicians and to assist efforts to increase the number of ratings on PRWs to consequently improve the fairness and practical importance of PRWs.

## Introduction

Although the increasing focus on evidence-based medicine and quality improvement has led to much progress, there remains significant unwarranted variation among the medical treatments that are routinely used in practice and deficiencies regarding all of the key aspects of high-quality health care [1-3]. However, potentially because of a lack of publicly available health care quality information, the members of the public are often unaware of such variations and quality differences [4].

Typically grounded in the assumptions of a theoretical consumer choice model [4], public-reporting activities have been developed with the aim of providing quality information about organizations or individuals to the public [5-8]. Public-reporting activities have two key aims: (1) influencing patient decision making by increasing the chance that the patients who obtain information will choose better quality organizations or individuals [4,9] and (2) driving quality improvement by identifying aspects of care needing improvement so that changes can be made in practice [4,9].

One type of public-reporting activity that has been developed in recent decades is physician rating websites (PRWs), which allows patients to anonymously rate, comment, and discuss physicians’ quality online as a source of information for others [10-13]. In addition to more than the 30 private PRWs internationally [14,15], an increasing number of public PRWs have been developed by governments and statutory health insurers. For instance, the United Kingdom launched the NHS Choices website in 2007 [16], which has evolved to allow patients to rate both physicians and hospitals, and Germany’s largest public health insurer, Allgemeine Ortskrankenkasse (AOK), launched a similar website called Arztnavigator in 2010, which was rolled out nationwide in May 2011 [17].

Medical association representatives, however, have often been strongly opposed to the development of PRWs, referring to them as a “meaningless popularity contest” and expressing concerns that PRWs would be used for “doctor bashing” or defamation [18,19]. For example, the president of the German Medical Association responded in 2009 with regard to the planned introduction of the Arztnavigator by AOK by criticizing the “Marketing Antics” of AOK, describing PRWs as “platforms for denunciation” [19]. Furthermore, a number of shortcomings of PRWs have been identified, including incomplete lists of physicians, low number of physicians rated, and low number of ratings per physician that are overwhelmingly positive, which in turn has raised concerns about the representativeness, validity, and usefulness of information on PRWs [15,20]. Indeed, recent research has indicated that PRWs can influence patient decision making and have an impact on quality improvement [21,22]; however, the ability of PRWs to achieve these goals is somewhat dependent on PRWs having a sufficient number of ratings.

At the beginning of 2010, a study was conducted to examine the evaluation criteria, evaluation tendencies, and utilization of German PRWs not only to allow a factual discussion of the current status quo of PRWs but also to serve as a baseline to document future developments and changes [23]. To examine the frequency of ratings and evaluation tendencies, a random stratified sample of 298 physicians from the physician associations in the states of Hamburg and Thuringia was generated and searched for on 6 German PRWs (Imedo, Jameda, Docinsider, Esando, Medführer, and Topmedic). It was reported that between 75% and 98% of selected physicians could be identified on one of the PRWs; between 3% and 28% of physicians had been rated at least once; the average number of ratings per physician ranged between 1.1 and 3.9; the maximum number of ratings per physician ranged from 3 to 27; and the average converted standardized rating (1=positive, 2=neutral, and 3=negative) ranged from 1.1 to 1.5 [23].

A number of other previous research studies have also examined the frequency of ratings and evaluation tendencies. In terms of the number of physicians rated at least once on PRWs, other previous studies in Germany have reported that between 3% and 26% of a sample of physicians had been rated in 2009 [24], 37% in 2013 [11], and 50% in 2014 [25]. In addition, previous studies conducted in the United States have reported that 16% of physicians were rated on RateMDs between 2005 and 2010 [26], and 27% of a sample of physicians had been rated in 2009 [15]. In terms of the average number of ratings per physician, other previous studies in Germany have reported an average number of ratings per physician of 2.8 in 2013 [11] and 3.1 in 2014 [25]. Research studies conducted in the United States have found a similar average number of ratings per physician: 2.4 [27], 3.2 [26], 2.4 [15], and 2.7 [28]. Finally, in terms of the average rating on PRWs, other previous German studies showed that almost 80% of all ratings on the PRW called “Jameda” were from the two best rating categories in 2013 [11], and 86% of the ratings on the 5 main PRWs were favorable (with 75% assigned to the best rating category and only 5% to the worst category) in 2014 [25]; an analysis of 3000 narrative comments on Jameda also found that 80% of all comments were positive [13]. Studies in the United States have produced similar positive results [15,26,27,29].

To examine the developments in the frequency of ratings and evaluation tendencies on German PRWs, the results of the 2010 study will serve as a baseline for the re-examination of the same 6 German PRWs. In addition, AOK-Arztnavigator was included in this study to assess how it compares with the other PRWs. The objectives of this study were therefore to examine (1) the number of identifiable physicians on German PRWs; (2) the number of rated physicians on German PRWs; (3) the average and maximum number of ratings per physician on German PRWs; (4) the average rating on German PRWs; (5) the website visitor ranking positions of German PRWs; and (6) how these data compare with 2010 results.

## Methods

### Sample

Following the 2010 study, a random stratified sample of physicians was generated from the physician associations in the German federal states of Hamburg and Thuringia. The state of Hamburg is a major port city in northern Germany and has a total population of 1,787,408 million residents (valid December 31, 2015; [30]) and a total of 15,831 physicians (valid December 31, 2015; [31]). The state of Thuringia lies in east-central Germany and has a total population of 2,154,816 million residents (1,091,735 million female; [32]) and a total of 12,530 physicians (valid December 31, 2015; [31]).

In October 2014, all physicians working in general medicine, obstetrics and gynecology, urology, and pediatrics were searched for on the websites of the Hamburg and Thuringia physician associations. From each specialty, a random sample was generated for each state, which comprised 50 physicians from general medicine, 33 physicians from obstetrics and gynecology, 33 physicians from pediatrics, and 33 physicians from urology. From the Thuringia physician association, the random sample comprised 50 of 976 general medical physicians, 33 of 289 obstetrics and gynecology physicians, 33 of 183 pediatric physicians, and 33 of 83 urology physicians. Therefore, the sample of 149 physicians selected for the study represented 9.7% of a total of 1531 physicians. From the Hamburg physician association, the random sample comprised 50 of 634 general medical physicians, 33 of 238 obstetrics and gynecology physicians, 33 of 123 pediatric physicians, and 33 of 71 urology physicians. Therefore, the sample of 149 physicians selected for the study represented 14% of a total of 1066 physicians.

The 6 PRWs (Imedo, Jameda, Docinsider, Esando, Medführer, and Topmedic) used in the 2010 examination were again selected to allow comparison. In addition, AOK-Arztnavigator was also included in this study to assess how it compared with the other PRWs. AOK, Germany’s largest public health insurer, launched Arztnavigator nationwide in May 2011 after the data collection of the initial study. Selected physicians were therefore searched for on a total of 7 PRWs: Imedo, Jameda, Docinsider, Esando, Medführer, Topmedic, and AOK-Arztnavigator.

### Data Collection

Between October and December 2014, every selected physician in the sample was searched for on the 7 PRWs. If a physician could not be found, this was recorded as “not found.” If a physician could be found, the physician’s rating and the number of ratings (if any) were recorded. On the PRW AOK-Arztnavigator, the results of the ratings are only published if there are at least five ratings. Consequently, data were recorded separately for physicians with more than 5 ratings and physicians with less than 5 ratings.

As the PRWs use different rating scales (percentage, school grade, and stars), the scales were recoded to standardize average ratings (see Table 1; [15,23]). Although recoding the rating scales results in a loss of richness, for reasons of comparability with the 2010 examination, this system was used again. However, to make the variation more transparent, original average ratings have also been listed.

Alexa Internet (www.alexa.com) was once again used to examine visitors to PRWs, compared with other websites. Founded in 1996, Alexa provides commercial Web traffic data and analytics. Traffic estimates are based on data from a global traffic panel and from websites that have chosen to install the Alexa script on their site and certify their metrics. The Alexa global traffic ranking is based on the estimated average of daily unique visitors and its estimated number of page views over the past 3 months relative to all other websites. In addition, Alexa provides a similar country-specific ranking, based on how a website ranks relative to other websites in a particular country over the past month [33]. The 7 PRWs were searched for on Alexa and their Germany-specific ranking recorded. Although AOK-Arztnavigator was not one of the PRWs examined in the first study in terms of frequency of ratings and evaluation tendencies, it was included in the first website visitor ranking table for comparison purposes.

**Table 1:** Recoding of original rating scales of physician rating websites (PRWs) to standardize ratings scale.

Physician rating websites and original rating scales		Recoding							
**Docinsider**									
	6 star rating	0 (− −)	1	2		3	4		5 (++)
	Recoding^a^	3	3	2		2	1		1
**Imedo and Esando**									
	5 star rating	1 (− −)	2	3		4	5 (++)		
	Recoding	3	3	2		1	1		
**Medführer and AOK-Arztnavigator**									
	Percent rating	0-33.3			33.3-66.6			66.6-100	
	Recoding	3		2			1		
**Jameda and Topmedic**									
	German school grade rating	1 (++)	2	3		4	5		6 (− −)
	Recoding	1	1	2		2	3		3

^a^Recoding: 1=positive, 2=neutral, and 3=negative.

### Data Analysis

All statistical analyses were conducted using Statistical Package for the Social Sciences (SPSS version 24 for Windows, IBM Corporation). Descriptive statistics included means and standard deviations for continuous variables and percentages for categorical variables. Relative change percentages were included for all variables with data from both studies. Two PRWs (Jameda and Docinsider) offer users two options to provide feedback, which include providing a rating (school grade or stars) or only recommending the physician. The number of these recommendations was assigned to the “number of ratings” and counted toward a positive rating. On the PRW AOK-Arztnavigator, physicians with less than 5 ratings have no published overall rating; the number of these ratings were recorded and counted toward “rated physicians” and “average number of ratings per physician.” To analyze differences between the two studies, chi-square tests were used for categorical data and *t* tests for continuously distributed data. The reanalysis of the 2010 data identified a number of minor errors in the results of the published 2010 study. These errors were corrected and data of this study compared with the corrected data rather than the published 2010 data.

## Results

Overall results combining both federal states are presented in Table 2. For transparency purposes, the results for each federal state are presented in Multimedia Appendices 1 and 2 (see Multimedia Appendix 1 for Thuringia results; see Multimedia Appendix 2 for Hamburg results).

### Identifiable Physicians

The proportion of physicians from the random sample that were able to be identified on the selected PRWs ranged between 65.1% (194/298) on Imedo to 94.6% (282/298) on AOK-Arztnavigator. This represents a decrease from the 2010 study, which ranged between 75.5% (225/298) on Medführer to 98.3% (293/298) on Jameda. Indeed, compared with the 2010 study, the portion of the physicians able to be identified significantly decreased on Imedo (χ^2^_1_=51 *P*<.001), Jameda (χ^2^_1_=27.3, *P*<.001), Docinsider (χ^2^_1_=9.4, *P*=.002), and Esando (χ^2^_1_=4.5, *P*=.03). However, the decrease of the overall portion of the sample (293/298, 98.3%) that was able to be identified on any of the PRWs compared with the 2010 sample (297/298, 99.7%) was insignificant (χ^2^_1_=2.7, *P*=.10).

### Rated Physicians

The proportion of physicians from the sample that had been rated at least once ranged between 16.4% (49/298) on Esando to 83.2% (248/298) on Jameda. This represents an increase from the 2010 study, which ranged between 3.3% (10/298) on Medführer to 27.8% (83/298) on Imedo. Indeed, compared with the 2010 study, the portion of the physicians that had been rated at least once increased on all PRWs, with the exception of Imedo, and very significantly so on Jameda (χ^2^_1_=191.4, *P*<.001), Docinsider (χ^2^_1_=17.8, *P*<.001), Medführer (χ^2^_1_=239.6, *P*<.001), and Topmedic (χ^2^_1_=46.1, *P*<.001). The increase of the overall portion of the sample (285/298, 95.6%) that had been rated at least once on any of the PRWs compared with the 2010 study (193/298, 64.7%) was also highly significant (χ^2^_1_=89.4, *P*<.001).

### Average and Maximum Number of Ratings

The average number of ratings per physician ranged between 1.2 (SD 0.5) on Esando to 7.5 (SD 6.7) on AOK-Arztnavigator. This represents an increase from the 2010 study, which ranged between 1.1 (SD 0.3) on Esando and 3.1 (SD 3.5) on Jameda for average number of ratings per physician. Indeed, all PRWs saw an increase in the average number of ratings per physician compared with the 2010 study, although the increase was found to be significant only for Medführer (*t*_12_=−10.5, *P*<.001, 95% CI −2.936 to −1.933) and Imedo (*t*_153_=−2.1, *P*=.04, 95% CI −0.722 to −0.021). However, the increase of the overall average number of ratings per physicians across all PRWs (5, SD 4.2) compared with the 2010 study (2.3, SD 2.8) was highly significant (*t*_476_=−8.4, *P*<.001, 95% CI −3.312 to −2.057). The aggregated average number of ratings per physician on all PRWs was 27.2 ratings, compared with 11.2 in 2010. This represents an average addition of 4 new ratings per physician each year on the German PRWs over 4 years. The maximum number of ratings per physicians ranged from 3 (Esando) to 115 (Docinsider). This represents an increase from the 2010 study, which found that the maximum number of ratings ranged from 2 (Esando) to 27 (Docinsider).

### Average Converted Standardized Rating

The average converted standardized rating (1=positive, 2=neutral, and 3=negative) ranged between 1.0 (SD 0.1) on Medführer to 1.2 (SD 0.4) on Jameda and Topmedic. This represents a further improvement toward “very good” from the 2010 study, which found a range between 1.1 (SD 0.4) on Imedo and Jameda to 1.6 (SD 0.7) on Medführer. Although the average converted rating improved on 4 PRWs (Docinsider, Esandoa, Medführer, and Topmedic) compared with the 2010 study, this improvement was significant only for Docinsider (*t*_105_=4.0, *P*<.001, 95% CI 0.179-0.538) and Medführer (*t*_9_=2.7, *P*=.03, 95% CI 0.089-1.090). Nevertheless, the improvement of the overall average converted rating across all PRWs (1.1, SD 0.2) compared with the 2010 study (1.2, SD 0.5) was highly significant (*t*_255_=3.4, *P*=.001, 95% CI 0.053-0.200).

### Website Visitor Ranking Positions

The visitor ranking positions of the selected PRWs in Germany on Alexa indicates that the use of such websites is not common, with only Jameda (position 317) and Medführer (position 9796) being placed among the top 10,000 visited websites in Germany (see Table 3). In comparison, the hotel rating site holidaycheck.de ranking position was 118, with google.de in position 1. Compared with baseline data, only Jameda and Topmedic increased their ranking position, with the rest being visited less.

**Table 2:** Overall ratings of physicians.

Overall ratings, N (%)=298/2597 (11)		Imedo^a^	Jameda^b^	Docinsider^c^	Esando^a^	Medführer^d^	Topmedic^b^	AOK-Arztnavigator^d,e^	Overall
**Identifiable physicians**									
	n (%)	194 (65.1)	260 (87.2)	229 (76.8)	234 (78.5)	231 (77.5)	281 (94.3)	282 (94.6)	293 (98.3)
	2010 Baseline (%)	267 (89.6)	293 (98.3)	258 (86.6)	254 (85.2)	225 (75.5)	271 (90.9)	N/A	297 (99.7)
	Relative change, %	−27	−11	−11	−8	3	4	N/A	−1
	Pearson chi-square tests	χ^2^_1_=51, *P*<.001	χ^2^_1_=27.3, *P*<.001	χ^2^_1_=9.4, *P*=.002	χ^2^_1_=4.5, *P*=.03	χ^2^_1_=0.3, *P*=.56	χ^2^_1_=2.5, *P*=.12	N/A	χ^2^_1_=2.7, *P*=.10
**Rated physicians**									
	n (%)	72 (24.2)	248 (83.2)	119 (39.9)	49 (16.4)	188 (63.1)	101 (33.9)	212 (71.1)	285 (95.6)
	2010 Baseline (%)	83 (27.8)	80 (26.8)	72 (24.2)	36 (12.1)	10 (3.4)	32 (10.7)	N/A	193 (64.8)
	Relative change, %	−13	210	65	36	1780	216	N/A	48
	Pearson chi-square tests	χ^2^_1_=1.1, *P*=.30	χ^2^_1_=191.4, *P*<.001	χ^2^_1_=17.8, *P*<.001	χ^2^_1_=2.3, *P*=.128	χ^2^_1_=239.6, *P*<.001	χ^2^_1_=46.1, *P*<.001	N/A	χ^2^_1_=89.4, *P*<.001
**Average number of ratings per physicians**									
	Mean (SD)	1.8 (1.1)	6.7 (8.1)	4.8 (12.4)	1.2 (0.5)	3.7 (1.2)	1.7 (1.0)	7.5 (6.7)	5.0 (4.2)
	2010 baseline (SD)	1.4 (1.1)	3.2 (3.5)	2.8 (3.6)	1.1 (0.3)	1.3 (0.7)	1.5 (1.0)	N/A	2.3 (2.8)
	Relative change, %	29	109	71	9	184	13	N/A	117
	*t* test	*t*_153_=−2.1, *P*=.04	*t*_197_=−1.1, *P*=.27	*t*_190_=−1.3, *P*=.20	*t*_83_=−1.0, *P*=.32	*t*_12_=−10.5, *P*<.001	*t*_134_=−0.9, *P*=.40	N/A	*t*_476_=−8.4, *P*<.001
	95% CI	−0.722 to 0.021	−4.381 to 1.214	−4.883 to 0.970	−0.264 to 0.086	−2.936 to −1.933	−0.571 to 0.225		−3.312 to −2.057
**Maximum number of ratings per physicians**									
	n	6	67	115	3	6	6	38	N/A
	2010 Baseline	7	18	27	2	3	6	N/A	N/A
	Relative change, %	−14	272	326	50	100	0	N/A	N/A
**Average rating converted^f^**									
	Mean (SD)	1.1 (0.4)	1.2 (0.4)	1.1 (0.4)	1.1. (0.5)	1.0 (0.1)	1.2. (0.4)	1.1 (0.4)	1.1 (0.2)
	2010 baseline (SD)	1.1 (0.4)	1.1 (0.4)	1.5 (0.7)	1.2 (0.5)	1.6 (0.7)	1.3 (0.5)		1.2 (0.5)
	Relative change, %	0	9	−27	−8	−38	−8	N/A	−8
	*t* test	*t*_153_=−0.5, *P*=.65	*t*_325_=−0.6, *P*=.53	*t*_105_=4.0, *P*<.001	*t*_84_=0.3, *P*=.80	*t*_9_=3.0, *P*=.03).	*t*_43_=1.3, *P*=.21	N/A	*t*_255_=3.4, *P*=.001
	95% CI	−0.152 to 0.095	−0.141 to 0.072	0.179 to 0.538	−0.180 to 0.234	0.089 to 1.090	−0.076 to 0.331	N/A	0.053 to 0.200
**Average rating original (SD)**		4.2 (0.7)	1.8 (1.0)	4.6 (0.9)	4.6 (0.9)	72 (6.2)	1.6 (0.9)	88 (15.1)	N/A

^a^1 to 5 star: 1 star worst rating, 5 stars best rating.

^b^School grade: 6 worst rating, 1 best rating.

^c^0 to 5 star: 0 star worst rating, 5 stars best rating.

^d^Percentage.

^e^No baseline data are given for AOK-Arztnavigator because it was not included in the first study.

^f^Recoding: 1=positive, 2=neutral, and 3=negative.

**Table 3:** Website visitor ranking positions.

Ranking	Imedo	Jameda	Docinsider	Esando	Topmedic	Medführer	AOK-Arztnavigator
Current ranking^a^	14,624	317	16,360	77,669	209,119	9796	52,925
Baseline ranking^b^	1472	1128	3073	8340	273,403	8340	38,407^c^
Relative change, %	893	−72	432	831	−24	18	38

^a^The ranking relates to Germany as on January 11, 2016.

^b^Values from first study as on March 7, 2011.

^c^Although AOK-Arztnavigator was not one of the PRWs examined in the first study, it was included in the website visitor ranking table for comparison purposes.

## Discussion

This update of the frequency of ratings and evaluation tendencies of German PRWs has resulted in two key findings. First, although there has been an overall increase in the average number per physician of ratings on German PRWs, this increase has not been even across the PRWs. Second, the average rating of physicians has shown further improvement toward “very good.”

### Number of Ratings

It is generally assumed that PRWs will only be helpful for users, and fair for those who are rated, if there are a high number of ratings [15,20]. The overall increase in the number of ratings on German PRWs since 2010, both in terms of the number of rated physicians and the average number of ratings per physician, is therefore a positive development and one that is consistent with previous studies in Germany.

In terms of the number of physicians rated at least once, between 16.4% (49/298) and 83.2% (248/298) of the sample had been rated at least once, compared with between 3.3% (10/298) and 27.8% (83/298) in 2010. Other previous German studies have reported that between 3% and 26% of physicians had been rated at least once in 2009 [24], 37% in 2012 [11], and 50% in 2014 [25]. Although it is difficult to directly compare these figures, given the different sampling and time frames used, they do suggest an upward trend and are generally higher than those reported internationally [15,26,34]. All PRWs in our study, except for Imedo, saw an increase in the proportion of physicians rated at least once. However, the increase in the proportion of rated physicians was not even across the PRWs, with Jameda (248/298, 83.2%), AOK-Arztnavigator (212/298, 71.1%), and Medführer (188/298, 63.1%) having more rated physicians compared with Docinsider (119/298, 40%), Topmedic (101/298, 33.9%), Imedo (72/298, 24.2%), and Esando (49/298, 16.4%). Furthermore, the overall proportion of the sample that had been rated at least once on any of the PRWs increased to 95.6% (285/298) from 64.8% (193/298) in 2010.

Similarly, in terms of the average number of ratings per physicians on German PRWs, physicians had an average number of ratings between 1.2 and 7.5, compared with 1.1 to 3.1 in 2010. Other previous German studies have reported average number of ratings per physicians of 2.4 in 2013 [11] and 3.1 in 2014 [25]. Research in the United States have found similar average number of ratings per physician on PRWs [15,26-28,34]. Whereas all PRWs in our study saw an increase in the average number of ratings per physician, this increase was not even across PRWs, with AOK-Arztnavigator (average 7.5 ratings), Jameda (average 6.7 ratings), Docinsider (average 4.8 ratings), and Medführer (average 3.7 ratings) having on average more ratings per physician than Imedo (average 1.8 ratings), Topmedic (average 1.7 ratings), and Esando (average 1.2 ratings).

It appears, therefore, that there is a need to differentiate German PRWs. Whereas Jameda was slightly ahead of others German PRWs in terms of the number of ratings in 2010, the field was reasonably equally subdivided between different PRWs. However, in the subsequent 4 years, there has been a development with Jameda and the new AOK-Arztnavigator in particular, highlighting an increase in ratings more than the other PRWs. It remains to be seen whether the other PRWs will be able to increase their number of ratings in the future. However, it is noticeable how quickly AOK, Germany’s largest public health insurer, has been able to establish AOK-Arztnavigator as one of the most used German PRWs since being introduced nationwide in May 2011. Two other large public health insurers, Techniker Krankenkasse (TK) and BARMER GEK, have also subsequently developed their own PRWs (TK-Ärzteführer and BARMER GEK-Arztnavi). AOK, TK, and BARMER GEK all utilize a central database known as “Weisse Liste,” recruiting ratings from their insurees via their own platforms but pooling these ratings on the shared Weisse Liste. So, if a patient rates a physician on AOK-Arztnavigator, this rating will also appear on TK-Ärzteführer. Future updates are needed to assess whether this practice may allow the public health insurers to take a bigger share of the PRW ratings away from their smaller private competitors.

Whereas the overall increase in the number of ratings on German PRWs suggests that the practical importance of PRWs is increasing, the relatively low number of physician ratings indicates that PRWs are still used very little in Germany for posting ratings on current physicians. Despite the focus on informed and autonomous patients and the relatively high use of comparative quality information concerning other consumer services and products [35], the German public seem to be rather reluctant in contributing to comparative quality information on health providers.

However, currently there is limited research examining the reasons why patients are not rating their physicians on PRWs, and more research is needed regarding this issue. A recently published study by Patel et al [36] explored patients’ views regarding rating general practitioners on PRWs, within the context of other feedback methods available in England. Participants reported that they would not leave feedback on PRWs because of accessibility issues, privacy and security concerns, and because they felt that feedback left on a website may be ignored [36]. Hanauer et al [37] also asked participants in their 2012 US study to consider the implications of leaving negative comments about a physician. Participants reported being concerned that their identity could be disclosed (34%), and that the physicians may take action against them for leaving negative comments (26%) [37].

### Average Rating

Whereas physician representatives were concerned before the implementation of PRWs that they would be primarily used for “doctor bashing” or defamation [18,19], these fears have proved to be unfounded. The average rating of physicians has further improved toward “very good,” with the average converted standardized rating (1=positive, 2=neutral, and 3=negative) ranging from 1.0 to 1.2, compared with 1.1 to 1.6 in 2010. Other previous research has also found that the majority of ratings are overwhelmingly positive. In Germany, 86% of the ratings of the 5 main German PRWs were favorable in 2014 [25], whereas an analysis of 3000 narrative comments on Jameda from 2014 also found that 80% of all comments were positive [13]. Studies in the United States have produced similar positive results [15,26,27,29,34]. Such overwhelmingly positive ratings, however, raise concerns about the representativeness, validity, and usefulness of PRWs [15,20].

Whereas some form of trust is essential in all social relationships, it is particularly important when one finds themselves dependent on others for their well-being. Indeed, the need for trust is arguably greater in the health care setting than many other areas of life because of the ineradicable imbalances of power, knowledge, and vulnerability found there [38]. Given their position in society, physicians are the recipients of not only public trust but also of a close interpersonal trust by patients, who enter into the physician-patient relationship with the expectation that physicians will act competently and dutifully. Patients’ willingness to disclose information about such a relationship is likely to be extremely low unless their expectations are far exceeded, or they feel that their trust has been violated in some way. Research concerning the rating of products on Amazon has reported such a “bimodal” trend, with “amateur reviewers” (those who review only occasionally) typically contributing a review only because of a strong reaction to a product either because they love it or hate it, and that for some, doing so is an almost a cathartic experience [39]. One would expect to see a similar trend on PRWs, and further research would be helpful to better understand why there are not more negative experiences reported on PRWs.

It is clear, however, that at least in some countries, the lack of negative reviews on PRWs is partly because of strict data protection laws and legal responses taken to such reviews by physicians and their advocacy groups [40]. Whereas most businesses (particularly small businesses such as physicians) are concerned about negative reviews and the impact these might have on their reputation, physicians have been particularly opposed to reviews of their services and often take negative reviews more personally than other business owners [41]. It has been argued that “medical narcissism” is a key reason that physicians find it so difficult to acknowledge and disclose medical errors, as such a disclosure can be too much of a challenge to their self-image of competence, control, and treatment-oriented focus [42]. A similar response may be a contributing factor behind many physicians’ unwillingness to accept negative reviews on PRWs and their efforts to prevent transparency of patient experiences and satisfaction with their performance. Critical reviews on PRWs, however, are a (usually anonymous) type of “patient complaints,” which are seen by many as an opportunity to learn and improve care [43]. Whereas there is evidence that some physicians do use reviews on PRWs to improve care [22], this opportunity is likely to be limited while patients are being encouraged not to post negative reviews on PRWs [12], and the negative reviews that are posted are legally challenged.

### Limitations

This study has a number of limitations that should be taken into account when interpreting the results. First, the selection of German PRWs was not exhaustive; consequently, some PRWs that have gained importance since the 2010 study may not have been taken into account. Second, the fact that the sample was only taken from 2 states in Germany limits the generalizability of the results. Results in Thuringia and Hamburg, however, were very similar, and we have no reason to suspect other states in Germany would be significantly different. Third, the development of the frequency of ratings and evaluation tendencies is not longitudinal, as the same sample of physicians was not used in both studies. Fourth, as the PRWs or Alexa.com were not webcited in either study when data were collected, this prevents the results from being reproduced. Finally, it was not controlled for the time frame in which ratings were allowed to be published.

### Conclusions

This update of the frequency of ratings and evaluation tendencies of German PRWs indicates that there has been an overall increase in the number of ratings on German PRWs, both in terms of the number of rated physicians and the average number of ratings per physician. This is a positive development and suggests that the practical importance of German PRWs is increasing. However, the overall average number of ratings per physician of all PRWs represents an average addition of only 4 new ratings per physician each year over the 4 years, which indicates that PRWs are still used very little in Germany for posting ratings on current physicians. However, without a higher number of ratings, the PRWs will continue to have a limited value. Further research is needed to identify barriers for patients to rate their physicians and to assist efforts to increase the number of ratings on PRWs, thereby improving the fairness and practical importance of PRWs. The increase in the number of ratings has also not been even across the PRWs. Given that physicians’ ratings are currently spread out across PRWs in an uneven manner, it would be advisable for users of PRWs to utilize a number of PRWs when searching for a new physician. The implementation of a website using “meta-crawling” to pool physicians’ ratings across all PRWs may also be a helpful addition to the field to allow users to easily see all of a physician’s ratings in one place. Future updates are also needed to assess whether the practice of using a central database may allow the public health insurers to take an even bigger share of the PRW ratings away from their smaller private competitors. However, if these smaller PRWs are unable to significantly increase their number of ratings in the future, consideration should be given to whether their continued existence in the German PRWs market is providing value or is, in fact, causing harm. Finally, the continued overwhelmingly positive ratings on German RWs have not allayed fears regarding the representativeness and validity of PRWs. Further research would be helpful to better understand why there are not more negative experiences reported on PRWs. Additionally, the medical profession itself should do more to ensure that patients are not being actively discouraged by physicians to post critical reviews, as they are a potentially important opportunity for physicians to learn and improve care.

## Appendix

Multimedia Appendix 1 Ratings of physicians in Thuringia.

Multimedia Appendix 2 Ratings of physicians in Hamburg.

## Abbreviations

AOK: Allgemeine Ortskrankenkasse
PRW: physician rating website
TK: Techniker Krankenkasse
