# Supplementary material for: Developments in the Frequency of Ratings and Evaluation Tendencies: A Review of German Physician Rating Websites
Source: J Med Internet Res. 2017 Aug 25;19(8):e299. doi: 10.2196/jmir.6599 (PMC5591403; doi:10.2196/jmir.6599)
Supplement: Multimedia Appendix 1 [file jmir_v19i8e299_app1.pdf]

Multimedia Appendix 1: Ratings of Physicians in Thuringia

N (%) = 149/1531 (10)	Imedo[a]	jameda[b]	Docinsider[c]	Esando[a]	Medführer[d]	Topmedic[b]	AOK-Arztnavigator[d,e]	Overall
Identifiable physicians (%)	92 (62)	131(88)	114 (77)	118 (79)	117 (79)	144 (97)	141 (95)	146 (98)
2010 Baseline (%)	132 (89)	148 (99)	128 (86)	128 (86)	109 (73)	138 (93)	NA	149 (100)
Relative Change	-30%	-11%	-11%	-8%	7%	4%	NA	-2%
Rated physicians (%)	33 (22)	125 (84)	38 (26)	28 (19)	100 (67)	40(27) 28	110 (74)	143 (96)
2010 Baseline (%)	47 (32)	54 (36)	25 (17)	22 (15)	5 (3)	(19)	NA	101(68)
Relative Change	-30%	131%	52%	27%	1900%	43%	NA	42%
Average number of ratings per physicians	1.9 (SD:1.2)	6.2 (SD:6.4)	2.3 (SD:1.9)	1.2 (SD:0.5)	3.7 (SD:1.2)	1.6 (SD:0.8)	8.3 (SD:7.1)	5.2 (SD:4.5)
2010 Baseline	1.4 (SD:0.7)	3.7 (SD:3.5)	1.2 (SD:0.5)	1.1 (SD:0.4)	1.4 (SD:0.9)	1.4 (SD:1.0)	NA	2.2 (SD:2.4)
Relative Change	36%	68%	92%	9%	164%	14%	NA	136%
Maximum number of ratings per physicians	6	46	11	3	6	5	38	NA
2010 Baseline	4	17	3	2	3	6	NA	NA
Relative Change	50%	170%	266%	50%	100%	-17%		
Average rating converted[f]	1.0 (SD:0.2)	1.2 (SD:0.4)	1.0 (SD:0.2)	1.1 (SD:0.3)	1.0 (SD:0.1)	1.1 (SD:0.4)	1.0 (SD:0.2)	1.1 (SD:0.2)
2010 Baseline	1.2 (SD:0.5)	1.2 (SD:04)	1.5 (SD:0.8)	1.3 (SD:0.6)	1.4 (SD:0.9)	1.3 (SD:0.5)	NA	1.2 (SD:0.5)
Relative Change	-17%	0	-33%	-15%	-29%	-15%	NA	-8%
Average rating original	4.4 (SD:0.6)	1.9 (SD:1.0)	4.7 (SD:0.5)	4.6 (SD:0.6)	72 (SD:8.1)	1.4 (SD:0.9)	91 (SD:11)	NA

[a] 1-5 Star: 1 star worst rating, 5 stars best rating.

[b] School Grade: 6 worst rating, 1 best rating.

[c] 0-5 Star: 0 star worst rating, 5 stars best rating.

[d] Percentage

[e] No baseline data are given AOK-Arztnavigator because it was not included in the first study.

[f] Recoding: 1=positive, 2=neutral, 3=negative
