# Supplementary material for: Developments in the Frequency of Ratings and Evaluation Tendencies: A Review of German Physician Rating Websites
Source: J Med Internet Res. 2017 Aug 25;19(8):e299. doi: 10.2196/jmir.6599 (PMC5591403; doi:10.2196/jmir.6599)
Supplement: Multimedia Appendix 2 [file jmir_v19i8e299_app2.pdf]

Multimedia Appendix 2: Ratings of Physicians in Hamburg

N (%) =149/1066 (14)	Imedo[a]	jameda[b]	Docinsider[c]	Esando[a]	Medführer[d]	Topmedic[b]	AOK-Arztnavigator[d,e]	Overall
Identifiable physicians (%)	102 (69)	129 (87)	115 (77)	116 (78)	114 (77)	137 (92)	141 (95)	147(98.7)
2010 Baseline (%)	135 (91)	145 (97)	130 (87)	126 (85)	116 (78)	133 (89)	NA[4]	148(99.3)
Relative Change	-24%	-11%	-12%	-8%	-0.2%	3%	NA	-0.7%
Rated physicians (%)	39 (26)	123 (83)	81 (54)	21 (14)	88 (59)	61 (41)	102 (68)	142 (95)
2010 Baseline (%)	36 (24)	26 (17)	47 (32)	14 (9)	5 (3)	4(3)	NA	92 (62)
Relative Change	24%	373%	72%	50%	1660%	1425%		54%
Average number of ratings per physicians	1.7 (SD:1.0)	6.9 (SD:9.1)	5.9 (SD:14.7)	1.2 (SD:0.5)	3.8 (SD:1.2)	1.7 (SD:1.1)	6.6 (SD:6.0)	4.8 (SD:3.8)
2010 Baseline	1.5 (SD:1.4)	2.2 (SD:3.3)	3.7 (SD:4.1)	1.1 (SD:0.3)	1.2 (SD:0.4)	2 (SD:1.2)	NA	2.4 (SD:3.3)
Relative Change	13%	214%	59%	9%	216%	-15%	NA	100%
Maximum number of ratings per physicians	5	67	115	3	6	6	25	NA
2010 Baseline	7	18	27	2	2	3	NA	NA
Relative Change	-29%	272%	325%	50%	200%	100%	NA	
Average rating converted[f]	1.2 (SD:0.5)	1.2 (SD:0.4)	1.1 (SD:0.5)	1.2 (SD:0.6)	1.0 (SD:0.1)	1.2 (SD:0.4)	1.2 (SD:0.5)	1.1)SD:0.3)
2010 Baseline	1.0 (SD:0.2)	1.2 (SD:0.4)	1.4 (SD:0.6)	1.0 (SD:0)	1.8 (SD:0.4)	1.3 (SD:0.5)	NA	1.3 (SD:0.5)
Relative Change	20%	0	-21%	20%	-44%	-8%	NA	-15%
Average rating original	4.1 (SD:0.8)	1.8 (SD:1.0)	4.4 (SD:1.0)	4.5 (SD:1.2)	72 (SD:2.6)	1.7 (SD:0.9)	83 (SD:19)	NA

[a] 1-5 Star: 1 star worst rating, 5 stars best rating.

[b] School Grade: 6 worst rating, 1 best rating.

[c] 0-5 Star: 0 star worst rating, 5 stars best rating.

[d] Percentage

[e] No baseline data are given AOK-Arztnavigator because it was not included in the first study.

[f] Recoding: 1=positive, 2=neutral, 3=negative
